# Supplementary material for: Competence for transcellular infection in the root cortex involves a post-replicative, cell-cycle exit decision in Medicago truncatula
Source: eLife. 2025 Jul 4;12:RP88588. doi: 10.7554/eLife.88588 (PMC12227204; doi:10.7554/eLife.88588)
Supplement: Figure 8—figure supplement 1—source data 1. [file elife-88588-fig8-figsupp1-data1.zip › Figure 8-Figure Supplement 1-Source Data 1/Figure 8-figure supplement 1-source data 1.pdf]

: cropped area

Trial 1 — Trial 2

Anti-GFP 1 2 3 4 1 2 4

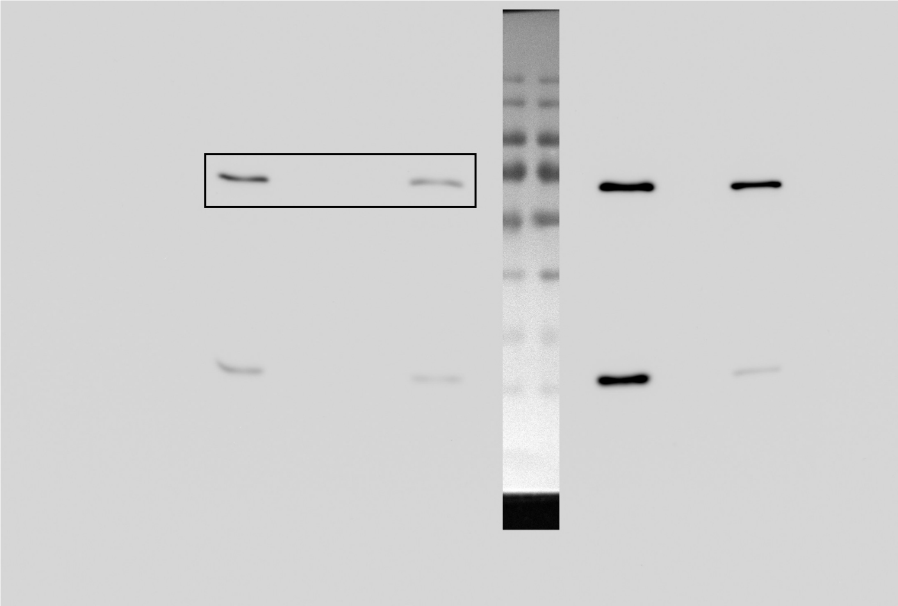

1: p35S::CYCD3;1-HA + pKNOLLE::eGFP-KNOLLE / pH3.3::H3.3-mCherry

2: pKNOLLE::eGFP-KNOLLE / pH3.3::H3.3-mCherry

3: pLjUbi::FLAG-NF-YA1 + pKNOLLE::eGFP-KNOLLE / pH3.3::H3.3-mCherry

4: p35S::CYCD3;1-HA / pLjUbi::FLAG-NF-YA1 + pKNOLLE::eGFP-KNOLLE / pH3.3::H3.3-mCherry

Trial 1 — Trial 2

Anti-DsRed 1 2 3 4 1 2 4

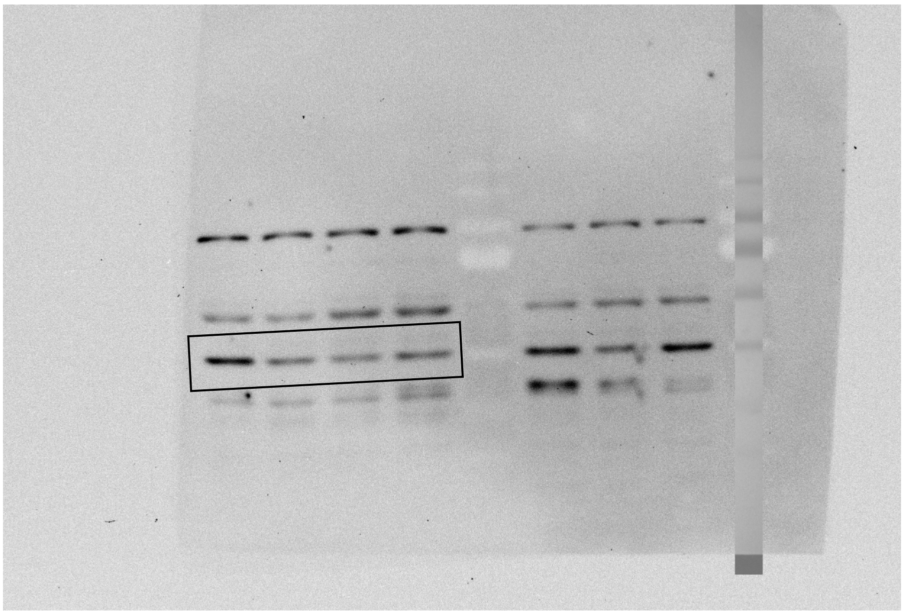

1: p35S::CYCD3;1-HA + pKNOLLE::eGFP-KNOLLE / pH3.3::H3.3-mCherry

2: pKNOLLE::eGFP-KNOLLE / pH3.3::H3.3-mCherry

3: pLjUbi::FLAG-NF-YA1 + pKNOLLE::eGFP-KNOLLE / pH3.3::H3.3-mCherry

4: p35S::CYCD3;1-HA / pLjUbi::FLAG-NF-YA1 + pKNOLLE::eGFP-KNOLLE / pH3.3::H3.3-mCherry

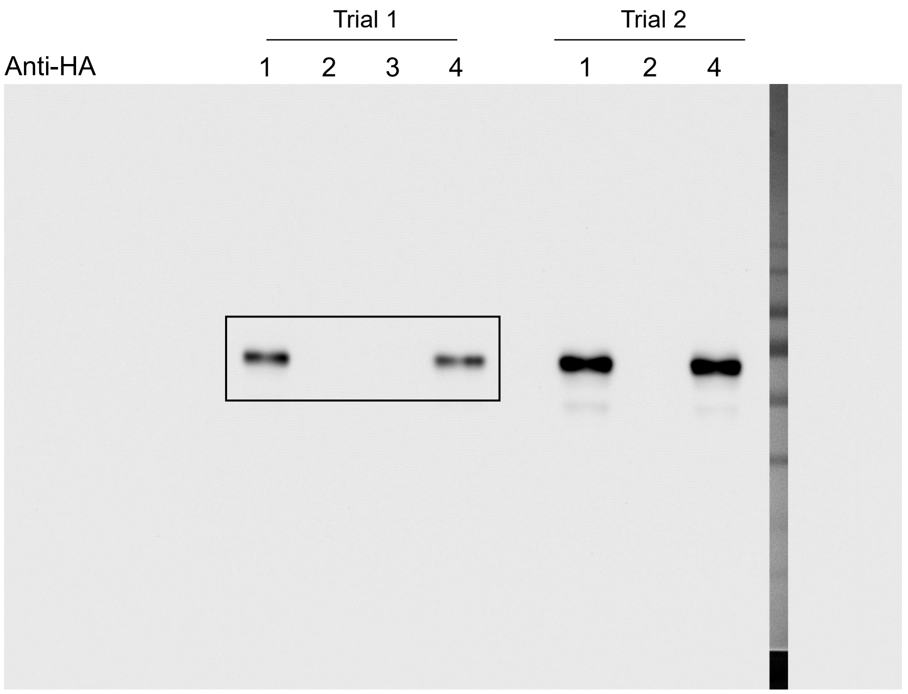

1: p35S::CYCD3;1-HA + pKNOLLE::eGFP-KNOLLE / pH3.3::H3.3-mCherry

2: pKNOLLE::eGFP-KNOLLE / pH3.3::H3.3-mCherry

3: pLjUbi::FLAG-NF-YA1 + pKNOLLE::eGFP-KNOLLE / pH3.3::H3.3-mCherry

4: p35S::CYCD3;1-HA / pLjUbi::FLAG-NF-YA1 + pKNOLLE::eGFP-KNOLLE / pH3.3::H3.3-mCherry

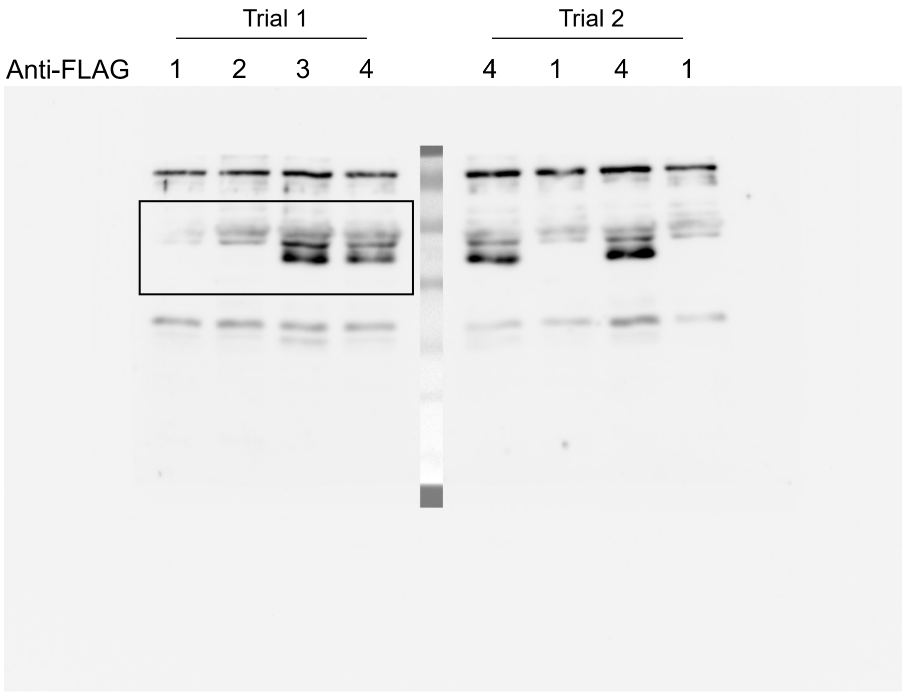

1: p35S::CYCD3;1-HA + pKNOLLE::eGFP-KNOLLE / pH3.3::H3.3-mCherry

2: pKNOLLE::eGFP-KNOLLE / pH3.3::H3.3-mCherry

3: pLjUbi::FLAG-NF-YA1 + pKNOLLE::eGFP-KNOLLE / pH3.3::H3.3-mCherry

4: p35S::CYCD3;1-HA / pLjUbi::FLAG-NF-YA1 + pKNOLLE::eGFP-KNOLLE / pH3.3::H3.3-mCherry

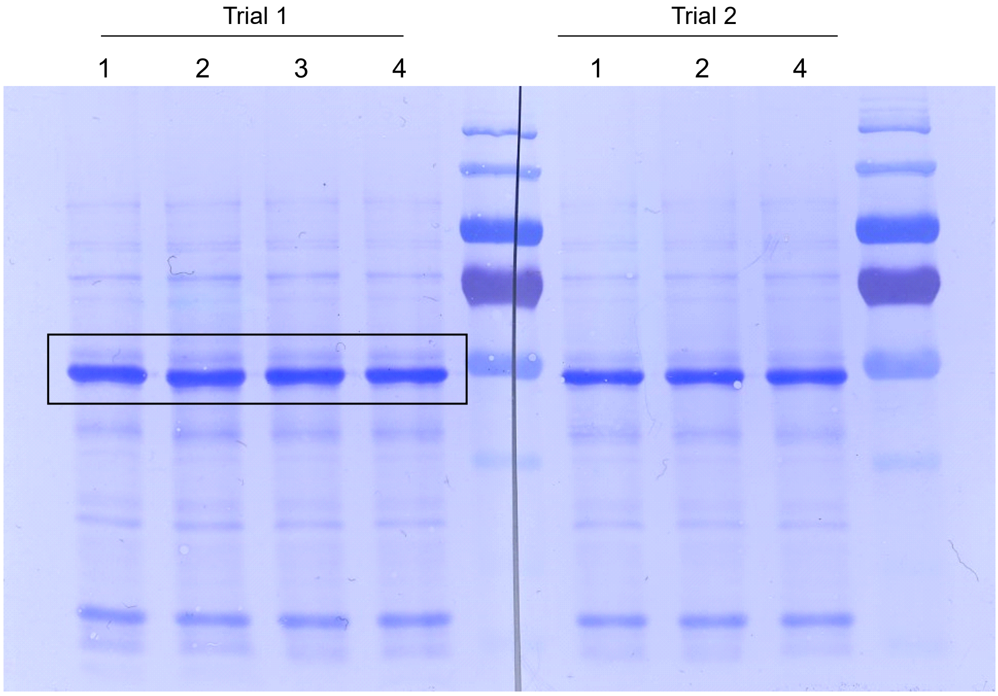

Coomassie blue-stained membrane (CBS)

1: p35S::CYCD3;1-HA + pKNOLLE::eGFP-KNOLLE / pH3.3::H3.3-mCherry

2: pKNOLLE::eGFP-KNOLLE / pH3.3::H3.3-mCherry

3: pLjUbi::FLAG-NF-YA1 + pKNOLLE::eGFP-KNOLLE / pH3.3::H3.3-mCherry

4: p35S::CYCD3;1-HA / pLjUbi::FLAG-NF-YA1 + pKNOLLE::eGFP-KNOLLE / pH3.3::H3.3-mCherry

Figure 8 – figure supplement 1 – source data 1. Original membranes corresponding to Figure 8 – figure supplement 1A. PageRuler™ Prestained Protein Ladder, 10 to 180 kDa was used (Thermo Fisher Scientific Product# 26616). Samples from 2 independent transformation experiments (Trial 1 and 2) are shown. Cropped areas displayed in Figure 8 – figure supplement 1A are indicated by a box.
