# Supplementary figures and images for: Competence for transcellular infection in the root cortex involves a post-replicative, cell-cycle exit decision in Medicago truncatula
Source: eLife. 2025 Jul 4;12:RP88588. doi: 10.7554/eLife.88588 (PMC12227204; doi:10.7554/eLife.88588)

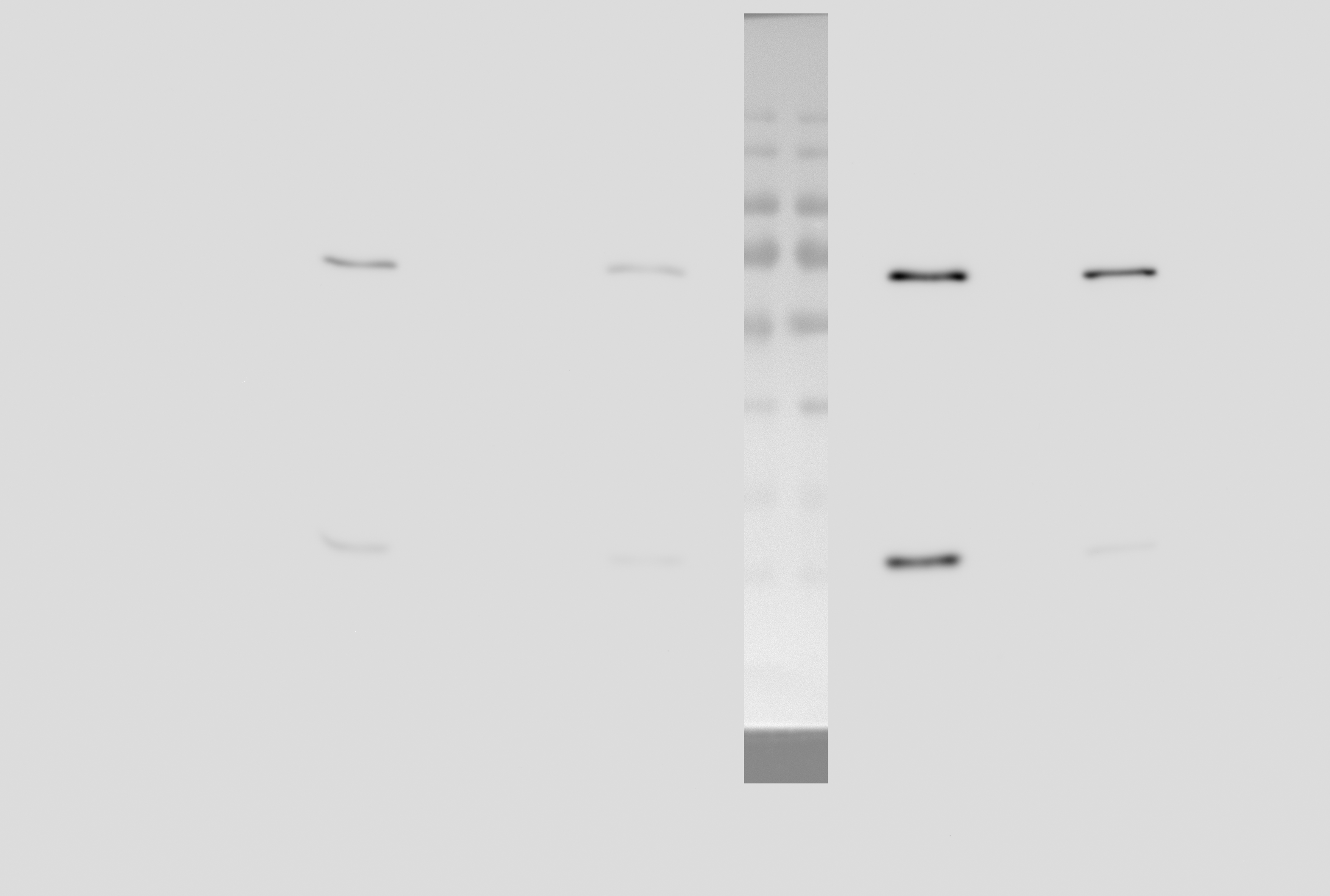

Supplement: Figure 8—figure supplement 1—source data 2. [file elife-88588-fig8-figsupp1-data2.zip › Figure 8-Figure Supplement 1-Source Data 2/Figure 8-Figure Supplement 1_A_1_KNOLLE.tif]

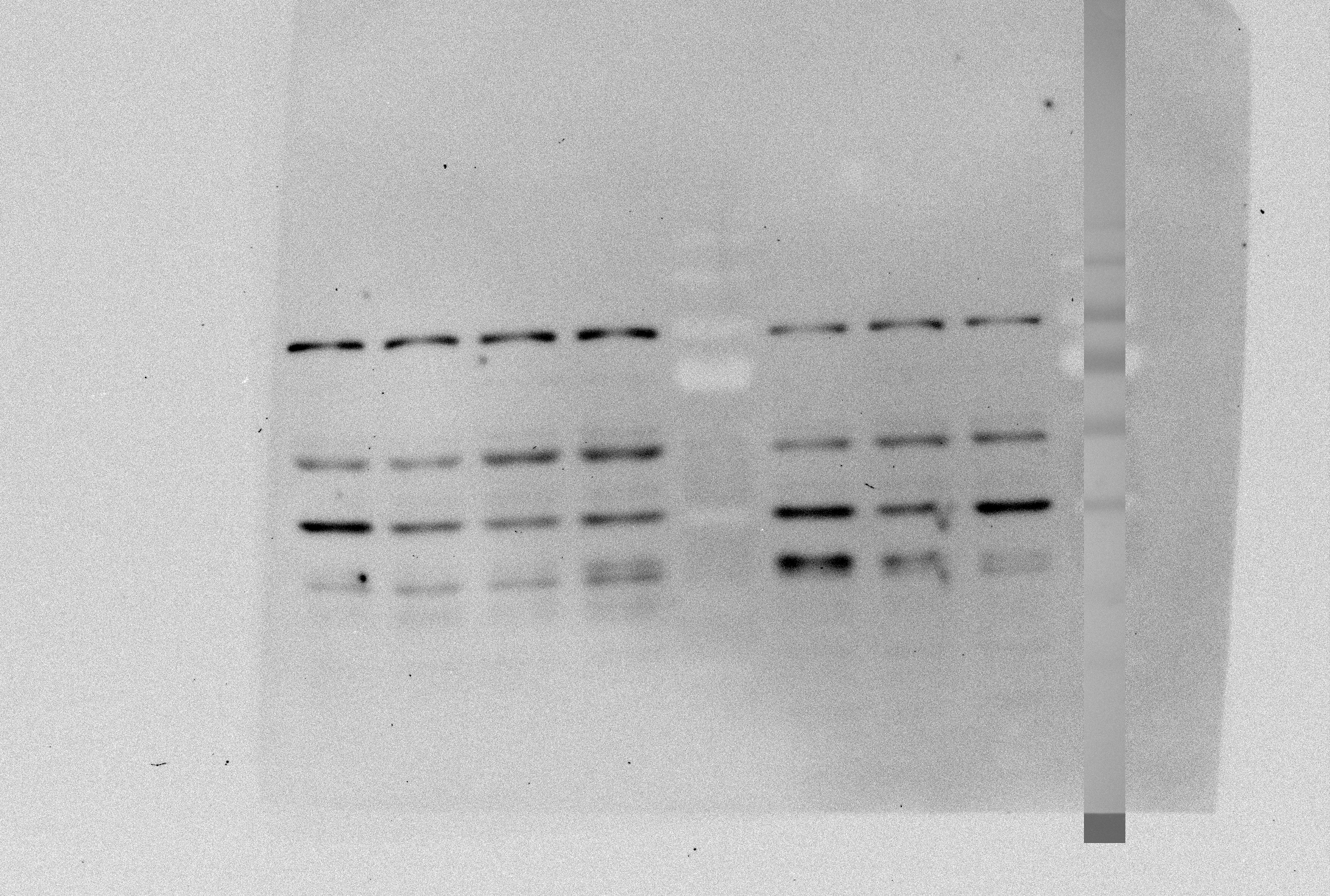

Supplement: Figure 8—figure supplement 1—source data 2. [file elife-88588-fig8-figsupp1-data2.zip › Figure 8-Figure Supplement 1-Source Data 2/Figure 8-Figure Supplement 1_A_2_H3-3.tif]

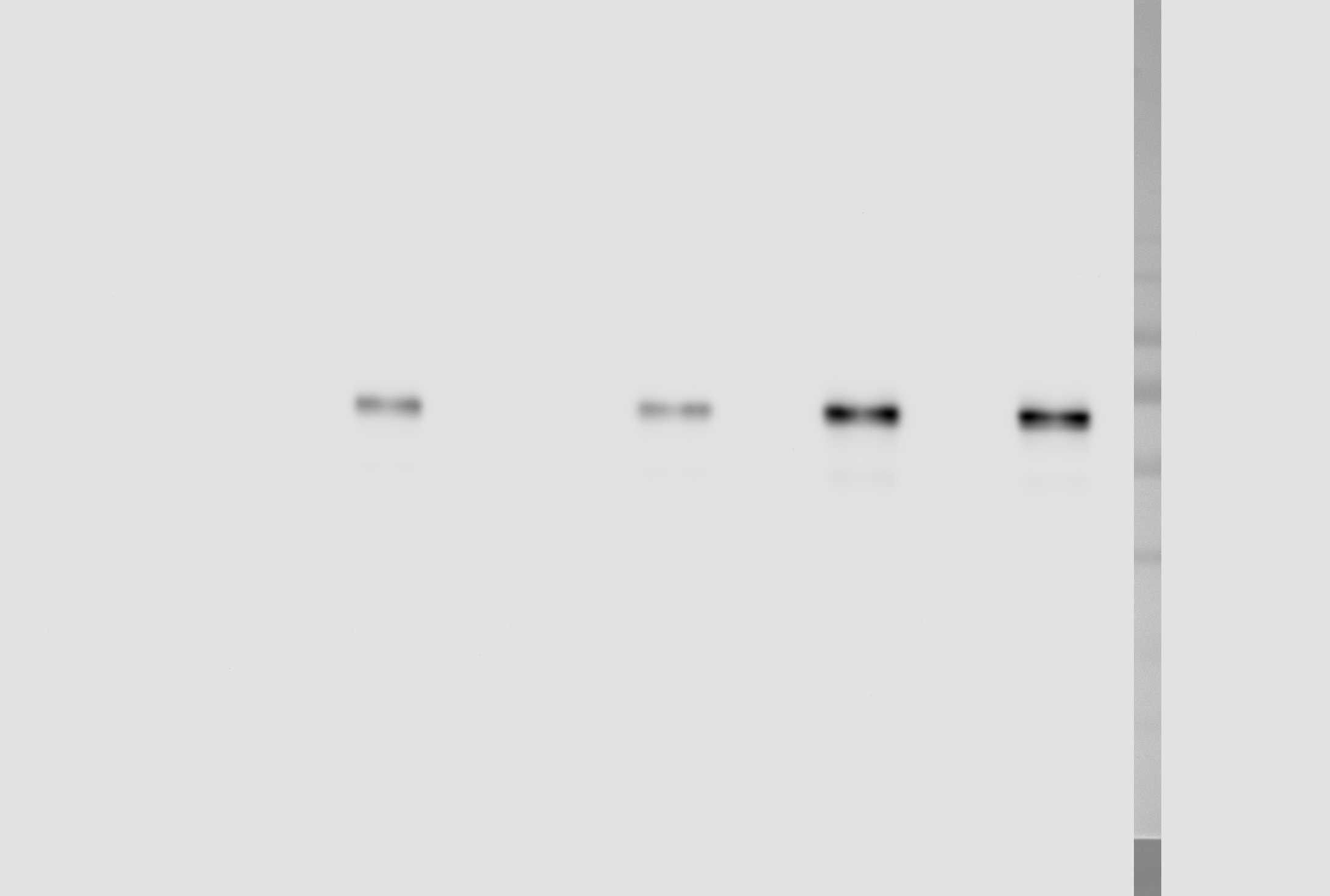

Supplement: Figure 8—figure supplement 1—source data 2. [file elife-88588-fig8-figsupp1-data2.zip › Figure 8-Figure Supplement 1-Source Data 2/Figure 8-Figure Supplement 1_A_3_CYCD3-1.tif]

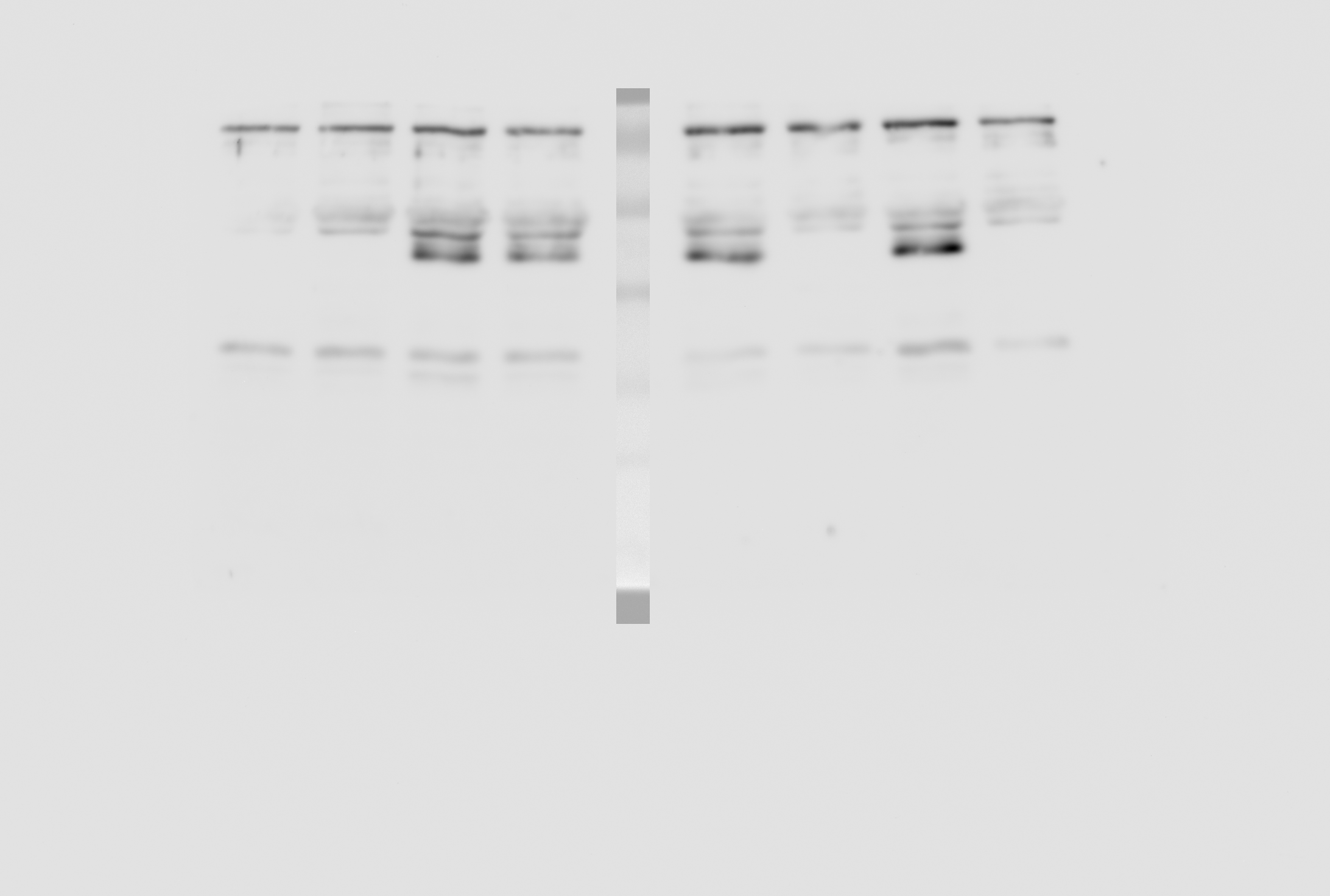

Supplement: Figure 8—figure supplement 1—source data 2. [file elife-88588-fig8-figsupp1-data2.zip › Figure 8-Figure Supplement 1-Source Data 2/Figure 8-Figure Supplement 1_A_4_NF-YA1.tif]

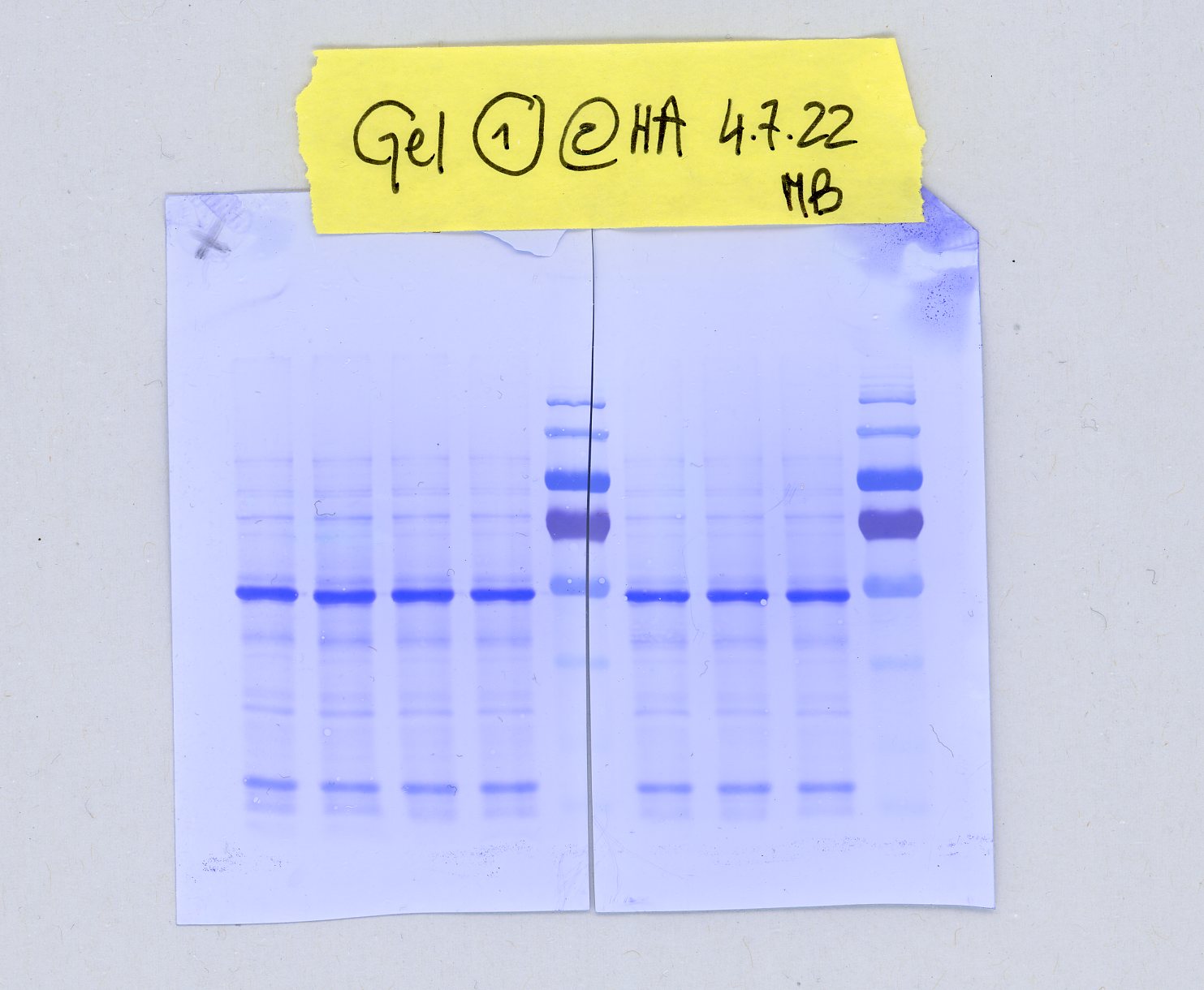

Supplement: Figure 8—figure supplement 1—source data 2. [file elife-88588-fig8-figsupp1-data2.zip › Figure 8-Figure Supplement 1-Source Data 2/Figure 8-Figure Supplement 1_A_5_CBS.jpg]
